# Contemporary insights into neuroimmune interactions across development and aging

**DOI:** 10.3389/fneur.2025.1611124

**Published:** 2025-07-25

**Authors:** Xin Yi Yeo, Yunseon Choi, Yeonhee Hong, Hyuk Nam Kwon, Sangyong Jung

**Affiliations:** ^1^Department of Medical Science, CHA University, Seongnam, Republic of Korea; ^2^School of Biological Science, University of Ulsan, Ulsan, Republic of Korea; ^3^Basic-Clinic Convergence Research Institute, University of Ulsan, Ulsan, Republic of Korea

**Keywords:** aging, neuroimmune crosstalk, immunosenescence, neurological decline, neurodegeneration

## Abstract

Initially considered distinct systems with independent physiological functions, recent evidence highlights the crucial role of active crosstalk between the nervous and immune systems in regulating critical physiological and neurological processes and immunological homeostasis. The identification of a direct body-brain circuitry allowing the monitoring of peripheral inflammatory responses, a unique skull bone marrow source of immune cells to the central nervous system (CNS), and the physical interface of the blood-brain barrier with the meningeal system suggest direct intersystem interactions, which can be further modulated by the local tissue environment, allowing non-neurological factors to influence neurological outcomes and vice versa. While there is a recognized age-dependent decline in both neurological and immune system function, in part due to the natural accumulation of cellular defects and the development of chronic systemic inflammation, it is unclear if the pre-existing bidirectional feedback mechanisms between the neurological and peripheral immune system plays a role in shaping the system decline, beyond commonly investigated pathological conditions. In this review, we will explore the effect of aging on the bidirectional communication between the neurological and immunological systems and attempt to understand how the inevitable age-dependent alterations of the interaction may concurrently drive immunosenescence, normal neurological decline, and neuropathological progression.

## 1 Introduction

Humankind has long pursued the goal of extending both lifespan and healthspan, often through lifestyle modifications, diet interventions, or natural remedies aimed at counteracting diseases (1, 2), despite a limited understanding of the underlying causes of human mortality. Systemic scientific investigation into the mechanisms driving the time-dependent decline in physiological integrity began only about half a century ago, initially focusing on non-mammalian organisms (3–6) and cancer models (7, 8), which revealed that lifespan is under polygenic control. In recent years, it has become increasingly apparent that aging outcomes can be driven independently or in combination by physiological and pathological degenerative processes. Physiological aging is a universal process characterized by the gradual accumulation of damage in cellular structures and repair mechanisms (9–12). In contrast, pathological aging shares many of the molecular pathways of physiological aging but is further influenced by genetic predispositions and environmental factors that accelerate the decline of specific organ systems (13, 14). A comprehensive review of the hallmarks of aging has been provided by López-Otín et al. (15, 16).

The immune system is among the first system hit by aging and its associated process. Following puberty, the thymus undergoes a natural involution, leading to a marked decline in the production of non-self-reactive naïve T cell and the reduced capacity to respond to novel antigens (17). Although the T cells maturation can occur in secondary lymphoid organs such as the spleen and lymph nodes (18, 19), or in response to environmental cues (20), this process is significantly impaired with age. Chronic infections further deplete the naïve T cell pool and promote the accumulation of senescent and exhausted T-cell clones (21). This immunosensence is accompanied by an increased risk of autoimmunity due to the expansion of self-reactive T cells (22) a shift of self-reactive CD8+ T cells toward innate-like immune responses (23), heightened pro-inflammatory activity from autoreactive T cells (24), and impaired immune regulation, partially due to reduced recruitment of functional capacity of regulatory T cells (25, 26).

Experimental studies often examine immune function in isolation, focusing on individual immune components or on the role of systemic or neuroinflammation in the development and progression of neuropathology (27). However, the nervous system itself is an underappreciated yet critical regulator of systemic immune responses. To comprehensively understand how aging impacts immune function, as well as how bidirectional communication between the immune and nervous systems contributes to neurological disease, it is essential to elucidate the role of the immune mediators in neural function.

## 2 The interdependence of the nervous and immune system development and function

### 2.1 Contribution of the primitive immune system to early central nervous system (CNS) development and function

Although the immune and nervous systems originate from distinct embryonic tissues (28, 29), they develop concurrently and exert reciprocal reciprocal influences on each other's basal functional capacities. The central nervous system (CNS) harbors resident immune cells, derived from peripheral sources, that are essential for maintaining normal neurological function throughout life. Hematopoiesis begins in the yolk sac, giving rise to immune cells with structural and physiological functions but limited cytotoxic potential compared to those generated in the bone marrow. Fetal natural killer (NK) cells are predominantly localized in the choroid plexus and meninges during development (30). Dysregulation of their activity has been linked to cerebral malformations, potentially mediated by pleiotrophin secreted by NK cells (30), which influence neural stem cell differentiation (31, 32), neurite outgrowth (33), and synaptic function (34). These fetal NK cells are rapidly depleted over time and replaced by bone marrow-derived NK cells, particularly under inflammatory and pathological conditions. In contrast, fetal mast cells enter the brain as early as embryonic day 12.5 (E12.5) in mice. The contribute to brain vascular remodeling (35) and hormone-dependent sexual differentiation of the brain (36, 37). Unlike NK cells, these mast cells persist into adulthood, within the brain's pia matter and thalamus (38, 39). They retain fetal-like properties and may contribute to physiological neuroimmune regulation in unknown ways.

**Figure 1:**
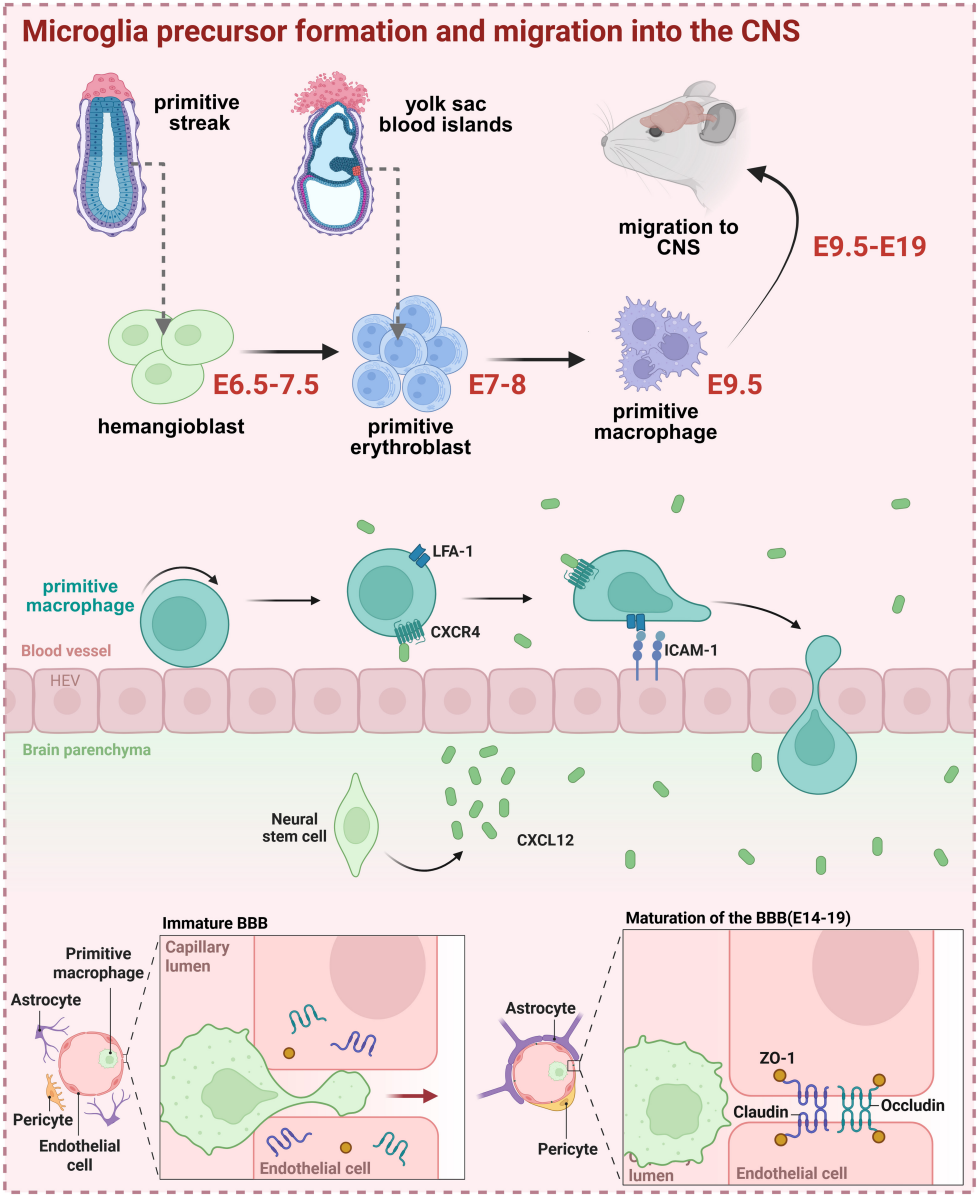
Microglia dynamics during brain development. Primitive macrophage, the precursors of microglia, originate from the yolk sac and enters the developing brain at approximately E9.5 **(top panel)**. These cells enter the developing brain primarily through the embryonic vasculature. The chemokine CXCL12, secreted by neural stem cells, guides the migration of primitive macrophages toward the interphase between the blood vasculature and the brain parenchyma. The lymphocyte function-associated antigen 1/Intercellular Adhesion Molecule-1 (LFA-1/ICAM-1) interaction is also proposed to facilitate trans-endothelial migration of these microglia precursors **(middle panel)**. As the BBB matures between E14 and E19, tight junctions formed between endothelial cells, and astrocyte endfeet along with pericytes engage the vasculature, to reinforce barrier integrity. This results in a selective permeable interface that restricts the entry of peripheral immune cells into the CNS **(bottom panel)**. Figure created with BioRender. Yeo, X. (2025) https://BioRender.com/uxni7uh.

The earliest major immune cell infiltration into the CNS occurs around E9.5 in mice, when erythromyeloid progenitor-derived primitive macrophages interact with fibronectin on embryonic blood vessels via α5β1 integrin receptors, guiding their migration into the developing brain through the pial surface and leptomeninges (40, 41). Ablation of sodium-calcium exchanger 1 (NCX1) results in defective circulatory development and the absence of primitive macrophages in the embryonic brain despite normal yolk sac haematopoiesis (42), suggesting that physical circulation is essential for their migration toward the CNS. Within the embryonic brain, local sources of colony-stimulating factor 1 (CSF1) and interleukin 34 (IL-34) are necessary to activate colony-stimulating factor 1 receptor (CSF1R) signaling in the infiltrating macrophages, promoting their proliferation and long-term maintenance in the CNS (43–46). Additionally, the interaction between C-X-C chemokine receptor 4(CXCR4) and its ligand CXCL12 directs immature macrophages toward the subventricular zone (SVZ) (47), where they engage with neural progenitors to modulate neurogenesis (48) (Figure 1). Transforming growth factor-β released by the neural precursors (NPC) further induces the expression of microglial identity genes (Sall1, Hexb, P2RY12), facilitating the differentiation of these primitive macrophages into microglia (49, 50).

The acquisition of microglia properties seems to be a largely context-specific phenomenon. Microglia retain several characteristics of peripheral macrophage, including their sensitivity toward cytokine and immune stimulus as well as their capacity to initiate immune responses in reaction to dynamic environmental conditions throughout life (51). The tightly regulated induction of programmed cell death in neural precursors (NPC) and newly generated neurons is essential for ensuring a quantitative match between the functional requirements of neuronal circuits and domains within the CNS (52, 53), and the elimination of excess or aberrant cells stochastically produced during the rapid process of neurogenesis (54). Microglia contribute to the pruning of NPCs and neurons through the release of proapoptotic factors that promote cell death via mechanisms independent of classical apoptosis (55, 56), caspase activation (57), excitotoxicity (58), or necroptosis (59). Damage-associated molecular patterns (DAMP) signals recruit microglia to the vicinity of aberrant cells, while additional molecular signals such as phosphatidylserine (54, 55) and calreticulin (55) mediate the phagocytic removal of damaged cells. Necroptotic microglia generated as a consequence of excessive phagocytic activity may themselves be removed by healthy microglia through C4b opsonization, thereby contributing to the maintenance of normal brain (56, 57).

Moreover, the release of purines, chemoattractant, and norepinephrine by neurons following changes in their activity (58), facilitates the redistribution of microglia within the brain parenchyma and supports microglia-dependent modulation of neuronal activity (59, 60). In a similar context, microglia play a pivotal role in the pruning and remodeling of synaptic contacts during neuronal circuit formation (61, 62). Disruption in microglia-neuron signaling between microglia and neurons that results in either excessive or insufficient synaptic pruning can lead to neuron structural dysfunction, a hallmark of various neurodevelopment and neurodegenerative disorders (63, 64). Despite substantial evidence linking altered microglial function to abnormal brain development (61, 65–67), a recent study employing a CNS-specific microglia ablation model suggests that certain microglia-dependent neurodevelopmental processes may proceed in their absence (68). These findings underscore the need to revisit and rigorously re-evaluate the established paradigm regarding the role of microglia in neurodevelopment and neuronal circuit formation.

The entry of peripheral immune cells into the CNS becomes increasingly restricted from mid-gestation (E14-E19 in mice). This transition is marked by a downregulation of microglia α5β1 integrin expression (40) and an upregulation of anti-migratory protein p27 (69), both of which significantly reduce the motility and the subsequent infiltration of microglia precursors into the developing brain after E13.5. In addition, the tight junctions begin to form between the claudin-5 and occludin molecules localized to the apical membranes of endothelial cells (70). Alongside the recruitment of pericytes and the extension of astrocyte endfeet, these events lead to the establishment of the selectively permeable blood-brain barrier that functionally isolates the CNS from the peripheral immune system (71, 72). This barrier effectively restricts further migration of peripheral immune cells into the parenchyma and confines resident microglia within the CNS (Figure 1). Inflammation events occurring before the complete formation of the BBB is thought to “prime” primitive microglia and other resident immune cells, inducing a persistent activation state. The early priming may alter microglial immunophenotypes and increase the population of resident microglia that is maintained into adulthood (73–75). Notably, a reservoir of cranial bone marrow-derived myeloid cell with immunoregulatory properties has been identified within the meningeal membrane (76, 77). These cells are situated adjacent to the glymphatic system, which serves as a conduit for the trafficking and interaction of peripheral immune cells with CNS-resident cell types (78). Intriguingly, immunogenic signals present in the cerebrospinal fluid (CSF) can be transmitted directly to the skull bone marrow, where they initiate local hematopoiesis before the activation of more distal sites such as the tibial marrow (79). The functional implication of this non-tibial immune cell source for the regulation and maintenance of neurological function remains largely unexplored.

### 2.2 Role of the immune system in peripheral nervous system (PNS) formation and the reciprocal role of the PNS for the CNS transmission of immune signals

**Figure 2:**
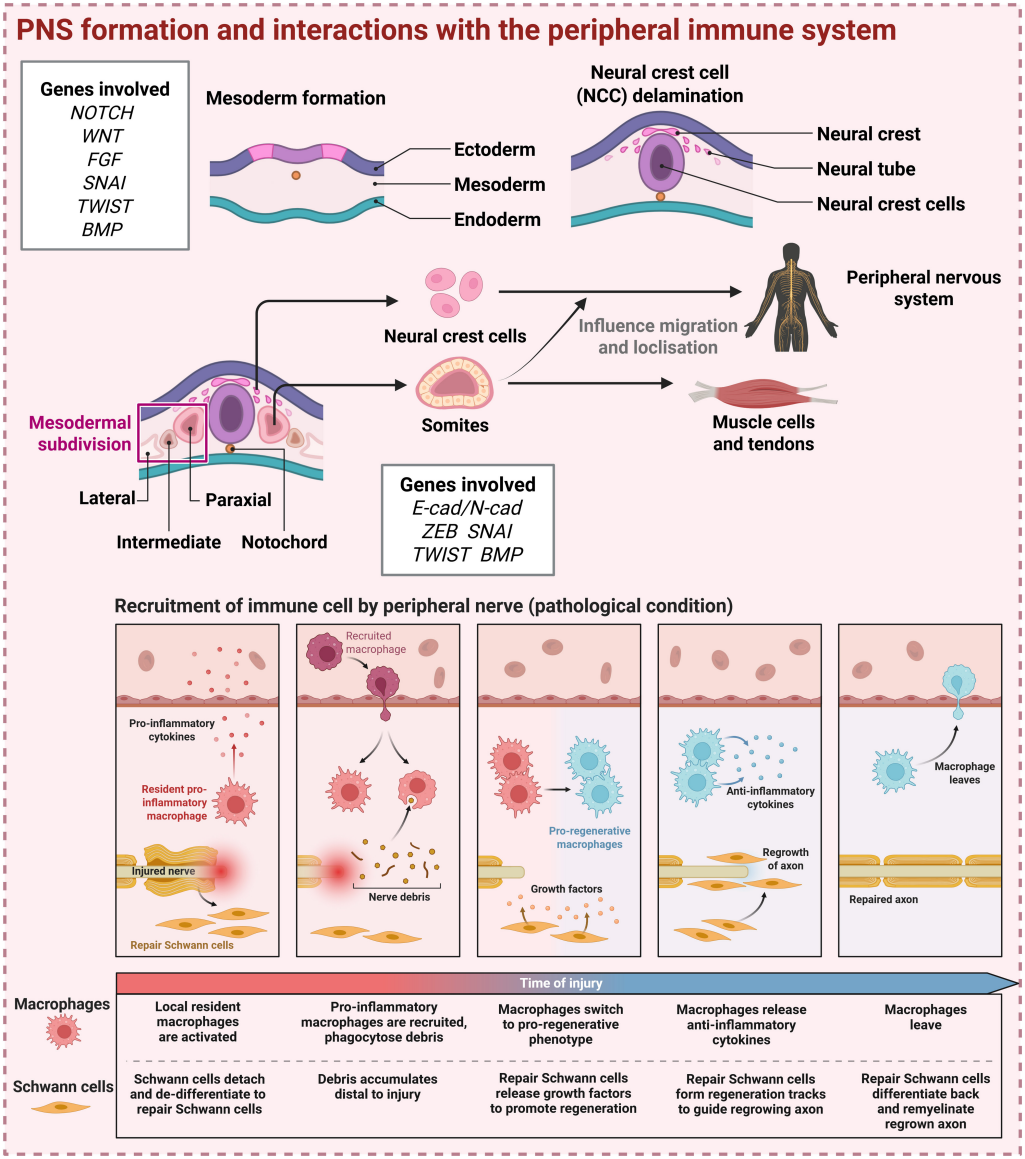
Development of the PNS and immune system influence on its function. Embryonic development of the PNS, originating from neural crest cells that migrate and differentiate intro diverse neuronal and glial populations along peripheral nerve tracts **(Top)**. Peripheral immune cells play a critical role in the regulation of PNS function and homeostasis. Although the contribution of immune cells to PNS development remains poorly understood, their involvement in peripheral nerve repair and regeneration following injury or under pathological systems is well-established **(Bottom)**. Created with BioRender. Yeo, X. (2025) https://BioRender.com/uxni7uh.

The PNS provides an alternative pathway for neuroimmune interactions as the CNS becomes increasingly restrictive to peripheral immune cell infiltration. Neural crest cells (NCC), the progenitors of neurons in the PNS (80), detach from the neural plate and migrate outward along developing peripheral nerve tracts following neural tube closure, a process regulated bytranscriptional and epigenetic mechanisms (81) (Figure 2, top section). NCCs differentiate into four functionally overlapping populations of cells arrayed along the anteroposterior axis of the embryo, distinguished by differentially HOX gene paralog expression that that determines the fate and localization of NCC derivatives (82, 83). For example, vagal NCC, located between somite 1 and 7, gives rise to the enteric nervous system (84) and contribute to the development of the heart (85, 86), thymus (87), and pancreatic ganglia (88). Conversely, sympathetic neurons originate from trunk NCCs situated between somite 6 and 17 along the spinal cord (82). The progressive radial migration of NCCs, utlising existing neurons as scaffolds, coupled with sequential fate-restriction influenced by environmental cues, facilitates the establishment of CNS control over distant organs such as the gastrointestinal tract (89).

Satellite glial cells derived from NCCs and residing in sensory and peripheral ganglia, are believed to function as resident immune-like cells, exhibiting macrophage-like properties including phagocytosis of cellular debris and pathogens (90). These cells express programmed death-ligand 1, which is critical for modulating surrounding T cell activity (91). Neuronal factors released by peripheral neurons modulate immune cell migration, activation, and local immune responses (92). In turn, immune cells proximal to the peripheral ganglia influence the development, maturation, and function of cytokine receptor-expressing peripheral neurons via cytokine signaling pathways (93, 94). An extensive review by Dr. von Andrian and his teamconsolidates current knowledge of the mechanisms underpinning peripheral neuroimmune interactions (95).

Within the PNS, neuropeptides play an important role in the modulation of tissue-resident immune cell function. Calcitonin gene-related peptide (CGRP) secreted by sensory TRPV1^+^ neurons upon detection of bacterial toxins, induces vasodilation, promotes keratinocyte proliferation to facilitate wound healing, and shapes immune responses by acting on Langerhans cells and dermal dendritic cells (96, 97). During *Candida albicans* skin infection, CGRPα stimulates IL-23 production by dermal dendritic cells, which in turn triggers IL-17A release from γδ T cells, thereby enhancing local antifungal immunity (98, 99). Simultaneously, CGRP reduces macrophage TNF-alpha production, inhibiting monocyte recruitment and preventing lymph node swelling (97). In allergic conditions such as those triggered by house dust mite exposure, peptidergic nociceptors release substance P, activating mast cells through Mas-related G-protein coupled receptor member B2 (MRGPRB2) signaling, and initiating allergic skin inflammation (100). TAFA chemokine-like family member 4 (TAFA4) produced by nociceptors promotes macrophage IL-10 secretion following ultraviolet-induced damage, supporting inflammation resolution and tissue repair (101). Nociceptors also regulate microfold (M) cell density and microbiota composition in the intestine to prevent pathogen invasion, with CGRP acting as a key modulator of these processes (102). Moreover, the Neuromedin U receptor signaling axis integrates enteric neuronal and innate immune responses to rapidly promote type 2 cytokine production, supporting tissue-protective immunity at mucosal surfaces (103, 104).

Afferent vagal neurons serve as a crucial communication pathway between peripheral immune cells and the brain, enabling the central nervous system to detect and respond to inflammatory signals. Broad vagal nerve stimulation has been shown to modulate systemic tumor necrosis factor α (TNF-α) levels following immune challenge (105, 106), suggesting the existence of an immunomodulatory network converging on the vagus nerve. Watkins et al. (107) further demonstrated that peripheral administration of the proinflammatory cytokine interleukin-1β (IL-1β) induces fever via vagal afferent pathways, highlighting the vagus nerve as a conduit for immune-to-brain signaling. The complex neuroimmunological effect of peripheral-derived cytokines, including their circulation, transport across the blood–brain barrier, and activation of circumventricular organs, requires careful consideration (108–110). A recently described body-brain neural circuit encompasses immune-stimulus-evoked cytokine productionand distinct vagal sensory neuron populations that selectively respond discretely to anti-inflammatory cytokines (TRPA1 expressing neurons) or pro-inflammatory cytokines (CALCA expressing neurons). The peripheral immune status can be adaptively regulated through transcriptomic reprogramming of the sensory neurons (111) or through acetylcholine-dependent suppression of proinflammatory cytokine production by macrophages by efferent vagal fibers (105). Moreover, these vagal neurons direct innervate dopamine β-hydroxylase-expression neurons within the caudal nucleus of the solitary tract in the brainstem, mediating peripheral-to-central immune signaling and restoring of immune homeostasis after immune activation (112). Furthermore, gut microbiota and their metabolites, including bile acid derivatives (113), can differentially influence vagal neuron activity (114, 115). Consequently, the status of the peripheral immune system is intricately linked to neurological function and underpins the neurobehavioral and cognitive outcomes observed in health and disease.

### 2.3 Potential sensitivity of the immune system to neurological modulation

The development of the CNS and PNS occurs in parallel with early hematopoietic waves in the fetal yolk sac and liver, during which precursors of key innate and adaptive immune cell lineages including macrophages, NK cells, B cells, and T cells are generated. These immune cells begin colonizing various organs early in development but generally acquire full functional competence only after birth. As gestation progressed toward term, the bone marrow gradually assumes the role of the primary site for immune cell production and replenishment.

Regardless of their anatomical origin or functional maturity, developing immune cells express a diverse array of neurotransmitter receptors, offering a mechanistic basis for nervous system influence over immune system maturation and function (see Table 1). However, due to on the predominant reliance on adult models in immunological research, and the fact that initial yolk sac and liver-derived primitive immune populations are largely supplanted by bone marrow-derived cells, our understanding of the specific neurotransmitter receptor expression profiles in the earliest waves of immune cells remains limited. Nevertheless, characterizing the receptor profiles and functional responses of adult immune cells to neurotransmitters can provide valuable insights into potential neural mechanisms for immune regulation. Such regulation is likely to be organ and niche specific. Mature immune cells are also capable of synthesizing and releasing neurotransmitters (116, 117), adding further complexity to the bidirectional communication between the nervous and immune systems and underscoring the integrated nature of neuroimmune regulation.

**Table 1:** The expression of neurotransmitter receptors and their potential role in the regulation of immune cell maturation and function.

**Cell type**	**Neurotransmitter receptors expressed**	**Potential effects of the activation of the neurotransmitter receptors**
Basophil	Nerve growth factor receptor, Trk (251)	Drives mediator release and primes basophils for C5a response (251)
	Acetylcholine receptor (aAChR) (252)	Regulate cell activation (253)
	Prostaglandin D receptor	Affects cell lifespan (254)
	Dopamine receptor (DRD5)	Inhibition of cell migration (255)
	Serotonin (5-HT) receptor (5-HT2B)	Downregulation of basophil-derived IL-4 (256)
	Adrenergic receptor (β2AR)	Affect basophil functional activity (257)
	GABAergic receptor	Inhibit degranulation in basophil (258)
B cells	Dopamine receptor (DRD1/DRD2-like) (259, 260)	Regulate cell migration (261), activation (262), and differentiation (259)
	Acetylcholine receptor (nAChR) (263)	Inhibit cell proliferation and antibody production (264), regulating the production of TNF-α, and decreasing B cell survival (265)
	5-HT receptor (266)	Increase mitogen-stimulated B-cell proliferation (267)
	Adrenergic receptor (β2AR) (268)	Activation enhance B cell receptor signaling, leading to the production of higher-affinity antibodies (269)
	GABA receptor (270)	Promotes germinal center B cell differentiation (270)
	Glutamatergic receptor (NMDAR)	Regulation of B cell migration and proliferation (271)
	Neuropeptide receptor	Modulate B cell activity (272)
Dendritic cell	5-HT receptor (273)	Inhibit proinflammatory cytokine and chemokine response (274), regulate migratory properties (275)
	Dopamine receptor D5 (DRD5) (276)	Required for LPS-induced IL-23 and IL-12 production (276)
	GABA receptor (277)	Enhance cell migration under pathological condition (277)
	Glutamatergic receptor	Involved in tumor-type-1 conventional dendritic cell crosstalk required to activate cytotoxic T cells (278)
	Neuropeptide (NK2R)	Activate dendritic cell-mediated type 1 immune responses (279)
	Adrenergic receptor (α1bAR)	Control cell migration (280)
	Acetylcholine receptor (mAChR)	Polarizes human dendritic cells toward a Th2-promoting profile (281)
Eosinophil	5-HT receptor (282)	Mediates chemotaxis and migration (283)
	Neuropeptide receptor (NK1R for Substance P) (284)	Activate cell and enhance cytotoxic granule release (284)
	GABA receptor (GABRA4)	Modulation eosinophil migration (285)
	Glutamatergic receptor (286)	–
	Adrenergic receptor (α1AR)	Modulation of eosinophil responses (287)
	Acetylcholine receptor (nAChR)	Down-regulate eosinophil function *in vitro* (288)
Mast cell	Acetylcholine receptor (nAChR) (252)	Influence degranulation and cytokine release (289, 290)
	Dopamine receptor (291)	D1-like receptor promotes degranulation in skin allergy model (292), D3 receptor suppress cell activation in rheumatoid arthritis (293)
	5-HT receptor (294)	Modulate chemotaxis (295)
	Neuropeptide receptor (296)	Activate cells, induce secretion of pro-inflammatory mediators (297)
	GABA receptor (lack γ subunit)	Suppress histamine release (258)
	Glutamatergic receptor (GluK2)	Involved in mast cell activation and degranulation (298)
	Adrenergic receptor (β2AR)	Inhibition of mast cell function (299)
	Cholinergic receptor	Induce degranulation and subsequent histamine release (300)
Monocyte/Macrophage	Dopamine receptor (DRD1/DRD2-like) (301, 302)	Macrophage polarization (301, 303), phagocytosis (304, 305)
	5-HT receptor (306)	Suppressed IFN-γ-induced antigen-presenting capacity (307)
	GABA receptor (308)	Promote monocyte differentiation into anti-inflammatory macrophages (309)
	Adrenergic receptors (α and β) (243)	Modulation of effector function (310, 311)
	Glutamatergic receptor (mGluR5)	Modulation of macrophage plasticity (312)
	Cholinergic receptor (nAChRα7)	Mediatiing macrophage recruitment to inflammaed sites (313)
	Neuropeptide receptors	Modulation of macrophage function (314)
Megakaryocytes (Erythro-myeloid progenitors, immune-modulatory)	Glutamate receptor (NMDAR) (315)	Proplatelet formation and maturation (316)
	Dopamine receptor (DRD1/DRD2-like) (317)	Induction of platelet production (318)
	Nicotinic acetylcholine receptor (nAChRα7) (319)	Inhibit megakaryopoiesis (320)
	5-HT receptor (317)	Promote megakaryopoiesis (321)
	GABA receptor (GABBR1) (322)	Regulate hematopoietic stem cell (HSC) proliferation, which may affect MK precursor production (323)
	Adrenergic receptor (α1AR) (324)	–
Neutrophils	Cholinergic receptor (325, 326)	nAChR inhibit TNF-α release and neutrophil recruitment during LPS-induced inflammation (325), M3 mAChR promotes neutrophil extracellular trap formation (326)
	Dopamine receptor (255)	Modulate cell function and apoptosis (255, 327)
	Neuropeptide receptor (328)	Modulate cell chemotaxis responses (329) and inflammatory status (330)
	Glutamate receptor (mGluR5)	Regulation of cell migration (331, 332)
	GABA receptor (GABABR)	Stimulation of neutrophil chemotaxis (333)
	Adrenergic receptor (β2AR)	Modulate neutrophil-specific effector functions (334)
	5-HT7 receptor (335)	–
NK cells	Cholinergic receptor (nAChRα7) (336)	Suppress pro-inflammatory cytokine release during autoimmune responses (336)
	Dopamine receptor (337)	Modulation of cellular cytotoxicity (337)
	Neuropeptide receptor (338)	Modulation of cell function (338)
	Glutamate receptor (mGluR5)	Modulation of IFN-γ production (339)
	GABA receptor (GABAAR)	Hampens NK cell cytotoxicity *in vitro* (340)
	Adrenergic receptor (β2AR)	Control adaptive NK cell response to viral infection (341), inhibit cell activity (342), affect cell circulation and adhesion (343)
	5-HT1A receptor	Regulate interaction between NK cell and monocyte (344)
Erythrocyte (Immune-modulatory)	Adrenergic receptor-like receptor (345)	Modulating the deformability of the cells (346)
	Glutamate receptor (NMDAR, NR2D and NR3B dominant) (347, 348)	Affect cell physical properties, oxidative state, and stability in circulation (348)
	Acetylcholine receptor	Affect membrane rigidity (349)
	Adrenergic receptor (β2AR) (350)	Modulate blood oxygen availability (351)
	Acetylcholine receptor (349)	Regulate self-renewal of early erythroid progenitors (352)
T cells	Adrenergic receptor (β2AR) (268)	Blocking β2-AR increases activation, proliferation, and cytokine release (353)
	Dopamine receptor (354–357)	Modulating the inflammatory status of CD4+ cells (356, 357), inhibit CD8+ cells (358), regulation of Treg response against self-antigens (359)
	Glutamate receptor (AMPAR) (360)	Important for CD8+ T cell cytotoxic function (361)
	5-HT receptor (362)	Enhancement of cell activation (363)
	Neuropeptide receptor (364, 365)	Sustain activated T cell survival (364), modulation of inflammatory response (365)
	Nicotinic acetylcholine receptor (nAChRα7)	Induce an increase in intracellular Ca^2+^ concentration (366)
	GABA receptor (GABAAR)	Inhibition of T cell proliferation (367)

While the physiological regulation of immune cell activation remains an area requiring further investigation, the influence of injury and pathology on immune recruitment and response is well-established. Peripheral nerve repair following injury involves a finely orchestrated immune recruitment and response that is essential for successful nerve regeneration (Figure 2, bottom). Immediately after injury, damaged axons and Schwann cells release DAMPs, which activate resident macrophages and Schwann cells via TLRs, triggering the production of pro-inflammatory cytokines and chemokines that facilitate immune cell recruitment (118, 119). Circulating monocytes are rapidly recruited to the injury site and differentiate into macrophages in response to local signals, including colony-stimulating factor 1 (CSF1) (120). Neutrophils also transiently infiltrate the injured nerve, contributing to initial myelin clearance but are quickly replaced by longer-lasting macrophages (121). These recruited macrophages undergo a phenotypic transition from a pro-inflammatory M1 state to an anti-inflammatory and pro-regenerative M2-like state, marked by expression of IL-10, arginase-1, and growth factors such as insulin-like growth factor-1 and vascular endothelial growth factor (122). This phenotypic shift is critical for resolving inflammation and facilitating regeneration. Peripheral inflammatory events can alter CNS activity through the activation and sensitization of nociceptors, which transmit signals to the spinal cord and higher brain centers involved in pain and stress regulation (123, 124). In response, descending modulatory pathways, particularly those originating from the periaqueductal gray and rostral ventromedial medulla, influence spinal nociceptive processing and autonomic outflow. These descending circuits can indirectly modulate peripheral immune function via sympathetic and parasympathetic outputs, including the release of neurotransmitters such as norepinephrine and acetylcholine, which act on immune cells to regulate inflammation (125, 126).

Adult hematopoietic homeostasis depends on the coordinated self-renewal, differentiation, and mobilization of HSC within the bone marrow microenvironment (127), and their recruitment to peripheral sites in response to inflammatory cues (128, 129). The sympathetic nervous system is the principal neural regulation of bone physiology, including remodeling a hematopoietic function. Direct sympathetic innervation originating from the thoracolumbar spinal cord preganglionic neurons extends into bone tissue (130). These nerve fibers are closely associated with blood vessels and reside in the hematopoietic cavities of the bone marrow, forming neurovascular units (131). This anatomical arrangement suggests that peripheral nerve signals influence bone and marrow-resident cells via diffusible chemical mediators. Disruption of β adrenergic receptor signaling has been shown to impair bone accrual (132), hinder hematopoietic regeneration (133), and reduce mesenchymal stem cell motility (134). In addition, circadian oscillations of adrenergic tone regulate the proliferation and cyclic release of hematopoietic cells (135, 136), largely through modulation of CXCL12 expression by stromal cells (137, 138). Sympathetic denervation dampens CXCL12 expression and significantly impaired HSC mobilization (139). Furthermore, the rhythmic and noradrenaline-dependent expression of endothelial adhesion molecules (140) emphasizes the role of adrenergic signaling and circadian timing in governing HSC trafficking and localization within the bone marrow niche. In contrast, the parasympathetic nervous system contributes choline acetyltransferase (ChAT)-positive fibers, likely originating from skeletal nerves (141), that have been implicated in linking physical activity to bone homeostasis. The precise role of parasympathetic innervation in the regulation of hematopoiesis remains undefined.

The establishment of multiple neuroimmune interaction nodes during development creates enduring sites for nervous system influence on immune regulation. Given the close physical and biochemical interactions between the immune cells and neurons, and their shared capacity to produce and respond to a common set of chemical messengers, age, and environmental-dependent alterations to the microenvironment inevitably the functionality of both systems. Therefore, the dynamic evolution of neural function and pathology must be interpreted in tandem with the context-dependent modulation of these neuroimmune interfaces.

## 3 Impact of aging on immune and nervous system function

### 3.1 Immunosenescence and inflammaging: mechanisms and consequences

The immune system undergoes a progressive functional decline with age, independent of the nervous system, characterized by both quantitative and qualitative shifts in innate and adaptive immunity. This decline compromises the body's ability to combat pathogens (142, 143). While the number of innate immune cells such as the macrophages (microglia in the CNS) and neutrophils remains relatively stable with age (144–146), their chemotactic and phagocytic capacity diminish (147) along with reduced cellular turnover (148). These defects result in increased accumulation of cellular debris (149) and impaired resolution of infections and inflammatory conditions (150, 151).

Age-associated alterations in cytokine production (152), toll-like receptor (TLR) signaling in response to pathogens (153), and impaired recruitment and migration of immune cells (154) further contribute to the state of persistent, low-grade immune activation that reflects a failed attempt to resolve chronic inflammation and infection. In parallel, thymic involution leads to marked reduction, and eventual cessation of naïve T cell production (155). Compounding this is the depletion of existing T cells due to repeated infections (156) and their subsequent clonal expansion and exhaustion, which attempts to compensate for impaired thymic output (157, 158). These factors contribute to telomere attrition and DNA damage, disrupting T cell homeostasis and survival (159, 160).

HSC in the aging bone marrow exhibit a skewed differentiation bias favoring myeloid over lymphoid lineages (161), coupled with reduced expression of activation-induced cytidine deaminase, an enzyme critical for antibody class switching (162). Consequently, aged individuals produced higher numbers of immature naïve B cells with diminished capacity to mount specific, long-term responses against novel antigens. In contrast, the adult skull bone marrow niche which is protected from systemic aging (163), and exhibiting differential responses to pathology compared to femoral bone marrow (164), may play a unique and as yet poorly understood role in CNS immune aging. The cumulation of these cellular and molecular changes in the peripheral immune system leads to immune competence, accumulation of tissue damage, increased risk of age-related complications, and elevated mortality.

Beyond impaired host defense, the aging immune system actively drives systemic aging. Chronically activated immune cells produce pro-inflammatory cytokines that damage tissues across multiple systems–including the nervous, musculoskeletal, and cardiovascular systems—via chronic inflammation of “inflammaging” (165). In the CNS, senescent microglia are implicated in neurodegeneration. These aged microglia display impaired clearance of protein aggregates (166), reduced motility, and compromised phagocytic activity, coinciding with a dystrophic morphology (167), loss of homeostatic gene expression (168), and metabolic shift toward fatty acid metabolism (169). The accumulation of lipid droplets further impairs debris clearance, including myelin remnants (170). Activated microglia induce a A1 phenotype in astrocytes via interleukin 1α (IL-1α), TNFα, and complement component (C1q) signaling (171), prompting astrocytes to secrete chemokines such as CXCL10 that attract T cells into the CNS through an increasingly permeable BBB (172, 173). This cascade sustains local neuroinflammation, activates resident glia, and promotes neuronal dysfunction and death.

In the musculoskeletal system, inflammaging disrupts the regulatory balance between interleukin 6 (IL-6) and myostatin, impairing the regenerative capacity of muscle satellite cells (174–176). IL-6 activates catabolic signaling via the Janus kinase (JAK)/signal transducer and activator of transcription 3 (STAT3) signaling pathway, inhibiting satellite cell differentiation and increasing myostatin expression (177), which in turn activates small mothers against decapentaplegic 2/3 (Smad2/3) signaling and induces cell cycle arrest (178). These changes reduce satellite cell proliferation, exacerbating sarcopenia and chronic inflammation (175, 179). In the adipose tissue, aging and metabolic dysfunction promote NOD-, LRR-, and pyrin domain-containing protein 3 (NLRP3) inflammasome activation (180), while targeting sirtuin 2, deacetylase regulating NLRP3, has been shown to reverse insulin resistance in aged mice (181). This highlights a tissue-specific interplay between immune dysfunction and metabolic disorders. Moreover, altered PNS function may modulate these immune responses, contributing to organ-specific degeneration and failure (182–184).

Recent studies have also highlighted the gut–immune–brain axis as a key player in immunosenescence and inflammaging (185–187). Aging-associated gut dysbiosis compromises intestinal immune homeostasis and increases gut permeability, facilitating the translocation of microbial products such as lipopolysaccharides (LPS) into systemic circulation (188, 189). Conversely, peripheral neuron activity may shape gut microbiota composition (190). These microbial-derived inflammatory cues perpatuate immune dysregulation. Interventions with probiotic and prebiotic aimed at restoring gut microbiota balance may mitigate this systemic inflammation (191). Collectively, these findings underscore the intricate interplay between immunosenescence, chronic inflammation, and systemic aging. They highlight the need for therapeutic strategies targeting immune rejuvenation to delay or prevent age-related diseases.

### 3.2 Impact of age-dependent systemic changes on neurological decline and neuropathology development

**Figure 3:**
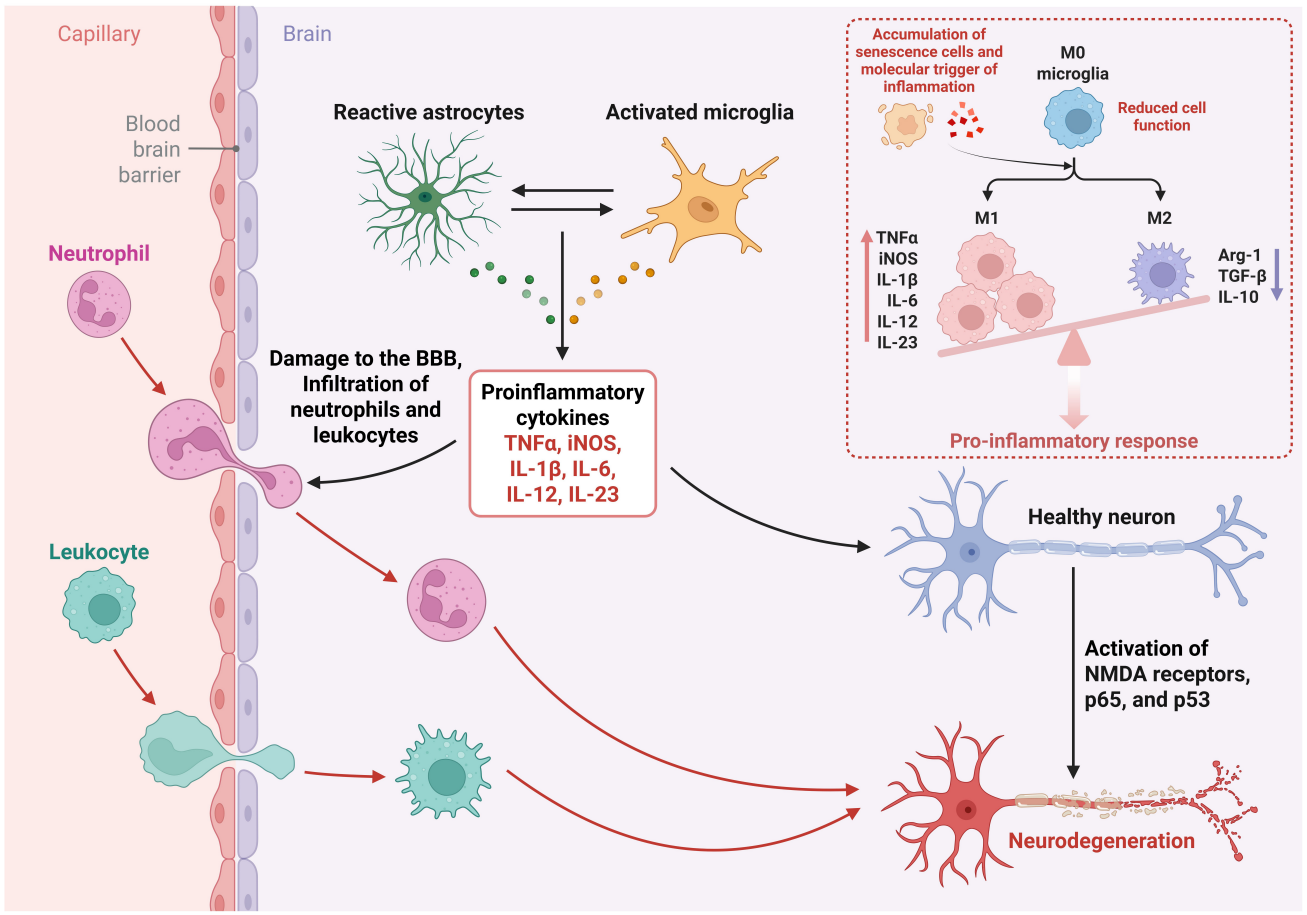
Age-related changes in immune cells and the development of chronic inflammation in the CNS. Increased permeability of the BBB due to endothelial cell, pericyte, and astrocyte senescence and demise and an increase in the number of activated astrocytes and microglia leads to the recruitment of peripheral immune cells into the brain parenchyma. The altered immune cell (from periphery or resident to the brain) function and status with age results in the accumulation of senescent cells, cellular debris, and abnormally aggregated proteins, triggering further inflammatory responses within the brain. As such, the persistent inflammatory status and increasingly abundant cellular byproducts result in a positive feedback loop that damages and triggers death pathways in neurons (neurodegeneration). Created in BioRender. Yeo, X. (2025) https://BioRender.com/dwabb60.

Endothelial cell senescence is a key contributor to age-associated BBB dysfunction. This process disrupts the formation of the endothelial glycocalyx (192) and downregulates the expression of tight junction proteins (193), leading to increased BBB permeability. The extent of BBB leakage is strongly correlated with changes in tight junction protein expression (194). Notably, the overexpression or pharmacological activation of silent information regulator 1 (Sirt1) has been shown to preserve BBB integrity in aging models, likely through attenuation of reactive oxygen species production and preservation of endothelial cell dysfunction (195, 196). Additional pathological changes, such as the accumulation of CNS-derived protein aggregates in pericytic (197), the loss of pericyte-astrocyte interactions (198, 199), impaired glymphatic waste clearance (200, 201), and sustained systemic inflammation (202). This disruption permits the infiltration of dysfunctional and pro-inflammatory peripheral immune cells into the CNS (203) (Figure 3). In synergy with chronically activated aged microglia and astrocytes, these infiltrating immune cells exacerbate neuroinflammation, compromise neuronal function, impair synapse maintenance, and potentiate the neurotoxic effects of abnormal protein aggregates, thereby accelerating neuropathological progression (139).

Cognitive decline in aging is further linked to persistent, low-grade neuroinflammation arising from complex bidirectional interactions between the CNS and the gut microbiota. Monocyte-driven gastrointesinal inflammation can increase gut permeability, enabling translocation of microbial products into circulation, which subsequently impacts the CNS (204). Aged mice exhibit elevated levels of circulating and brain-associated lipopolysaccharide (LPS), along with increased expression of Toll-like receptor 4 (TLR4), myeloid differentiation protein-88 (MyD88), and nuclear translocation of NF-κB in both intestinal and brain tissues (205). Moreover, Microbiome gut microbiota-derived short-chain fatty acids and metabolites such as 3-indoxyl sulfate can stimulate vagal nerve and NST activity (206), potentially modulating systemic and central inflammation via vagal pathways. The exacerbation of motor deficits in α-synuclein-expressing mice following fecal microbiota transplantation from Parkinson's disease (PD) patients suggests a potent gene-environment interaction in neurodegenerative disease pathogenesis (207).

Systemic metabolic and hormonal alterations further compromise neural function with age. Immune-metabolic crosstalk and cytokine-mediated interference in metabolic regulation contribute to the development of insulin resistance (208), ectopic lipid deposition (209, 210), and hypertension (211). Each of these factors independently heightens the risk for neuronal death and cognitive impairment (212–214). The decline in hormone levels with age further exacerbates deficits in glucose metabolism and sensing (215, 216) and inflammaging worsens existing defects in glucose metabolism and sensing (217). Of the earliest detactable changes in this cascade is the downregulation of glucose transporter type 4 (Glut4) expression in insulin-sensitive neurons (218, 219), which compromises synaptic energy supply (220) leading to cognitive dysfunction. Chronic hyperglycaemia also promotes tau hyperphosphorylation, a hallmark of Alzheimer's disease (221). In comparison, hypertension and dyslipidemia impair cerebral blood flow, increasing the risk of hypoperfusion-induced microinfarcts (222). Comprehensive investigation of the interplay between immune dysregulation, metabolic dysfunction, and neural decline is essential to delineate the mechanisms driving age-related neuropathology.

## 4 Modulation of the neuroimmune axis holds promise for the management and treatment of neurological pathology

Targeting the neuroimmune axis presents a promising approach for the treatment of neurological disorders. One such strategy involves the clearance of senescent immune cells using senolytic agents, which has demonstrated neuroprotective effects. The elimination of senescent immune cells with the use of senolytics has been shown to mitigate neurological decline by enhancing neuronal survival toward physical insults (223), reducing proinflammatory cytokine production (224) and abnormal protein aggregation (225) in the presence of neuroinflammation. As brain penetrant and non-penetrant senolytics are equally effective in reducing Ad pathology, the locus and mechanism of effect are unclear (226). Yet, care is required for the use of senolytics in the management of immune-related conditions with their potential for off-target toxicity (227). The heterogeneity of cellular senescence (228, 229) and specificity of senolytics to survival pathways also meant that there is no universal senolytic to clear all senescence cells and the potential unwanted removal of senescent, non-replaceable neurons may exert more harm to neurological function and neurocognitive outcomes.

Alternatively, anti-inflammatory therapies targeting neuroinflammation, a key driver of neurodegeneration can be achieved by modulating microglia activation (230), reducing prostaglandin-mediated inflammation (231), and inhibiting the complement system (232). The long-term use of non-steroidal anti-inflammatory drugs (NSAID) that target cyclooxygenases (COX) and the production of prostaglandin (233) is associated with a significant decrease in the risk of developing AD (234). Consistent with the observation, COX-2 inhibition prevents progressive degeneration of dopaminergic neurons in a preclinical model of Parkinson's disease (PD) (235). The inhibition of NLRP3 inflammasome with mefenamic acid and the complement pathway with anti-complement drugs have ameliorated amyloid beta deposition, synapse loss, and neuronal loss, and improved neurocognitive outcomes of genetic models of neurodegenerative disease (236, 237). The chronic use of NSAID risks gastrointestinal and renal toxicity (238, 239) while general inhibition of inflammation is likely effective only pre-symptomatically.

Glucagon-like peptide 1 (GLP1) agonist exerts a pleiotropic effect in the CNS to reduce inflammation and abnormal protein aggregation. Liraglutide treatment significantly reduced inflammation in the cortex of the APP/PS1 mouse model of AD (240) while Exenatide reduced TNFα expression and hippocampal neuron loss in a streptozotocin model of AD (241). On the other hand, GLP1 agonists may enhance autophagy and Aβ plaque clearance (242), improve brain insulin sensitivity and availability of glucose to neurons (243), and boost brain-derived neurotrophic factor (BDNF) signaling (244) to increase the chance for neuron survival in the presence of neuroinflammation and toxic protein aggregates. The time and dose of GLP1 administered is important for the greatest efficacy in the management of neurological conditions and the co-administration with anti-amyloid drugs may enhance the neuroprotective effect of GLP1 agonists in neurodegenerative diseases.

Given the absence of a modifying treatment for neurodegenerative diseases, lifestyle factors are an appealing strategy to manage progressive neurocognitive decline. Lifestyle changes have been linked to better cognitive functions in older individuals (245). Despite the difference in targets, common dietary interventions that limit saturated fats and processed food consumption (Mediterranean diet), induce ketosis (ketogenic diet), and restrict energy consumption (caloric restriction or intermittent fasting) aimed to increase the availability of the precursors essential for cellular recovery, reduce factors inflicted in cellular death in neurodegenerative conditions, and enhance autophagy to promote the clearance of protein aggregates (246, 247). Optimal diets vary greatly depending on the underlying genetics and disease stage of an individual and long-term adherence to a restrictive diet is challenging. The adoption of physical activity in various modalities is capable of slowing cognitive decline in patients with mild cognitive impairment and AD (248) through the expression of BDNF (249) and alleviation of neuroinflammation (250).

## 5 Conclusion

Modulating neuroimmune interactions offers a compelling strategy for the treatment of diverse neurological pathologies through the alteration of disease trajectories, alleviation of symptoms, and improving quality of life. Nonetheless, the heterogeneity of neuroimmune responses and disease status across individuals complicates treatment development. General immunosuppression carries risks of infection and malignancy while CNS-targeted therapies need to cross the BBB, and a delicate tuning of immune suppression is essential to maintain key immune functions while alleviating neurological defects. The development of reliable biomarkers to stratify patients, monitor neuroimmune activity, and assess therapeutic response is essential to the implementation of precision medicine approaches. Advances in nanotechnology and drug delivery systems may also enhance the precision and safety of neuroimmune-targeting interventions. Ultimately, the successful translation of neuroimmune modulation into clinical practice will depend on sustained interdisciplinary research. Collaborative efforts integrating immunology, neuroscience, metabolism, pharmacology, and systems biology are essential to unravel the complex interplay between systemic aging and neurological decline. By deepening our understanding of the neuroimmune axis, it may be possible to identify novel therapeutic targets and intervention windows that can halt or even reverse the progression of neurodegenerative diseases, offering hope for effective and individualized treatments in the aging population.
